# Point and trend accuracy of a continuous intravenous microdialysis-based glucose-monitoring device in critically ill patients: a prospective study

**DOI:** 10.1186/s13613-016-0171-3

**Published:** 2016-07-19

**Authors:** J. H. Leopold, R. T. M. van Hooijdonk, M. Boshuizen, T. Winters, L. D. Bos, A. Abu-Hanna, A. M. T. Hoek, J. C. Fischer, E. C. van Dongen-Lases, M. J. Schultz

**Affiliations:** Department of Intensive Care, Academic Medical Center, Room C3-311, Meibergdreef 9, 1105 AZ Amsterdam, The Netherlands; Department of Medical Informatics, Academic Medical Center, Amsterdam, The Netherlands; Department of Clinical Chemistry, Academic Medical Center, Amsterdam, The Netherlands; Laboratory of Experimental Intensive care and Anesthesiology (L.E.I.C.A), Academic Medical Center, Amsterdam, The Netherlands

## Abstract

**Background:**

Microdialysis is a well-established technology that can be used for continuous blood glucose monitoring. We determined point and trend accuracy, and reliability of a microdialysis-based continuous blood glucose-monitoring device (EIRUS^®^) in critically ill patients.

**Methods:**

Prospective study involving patients with an expected intensive care unit stay of ≥48 h. Every 15 min, device readings were compared with blood glucose values measured in arterial blood during blocks of 8 h per day for a maximum of 3 days. The Clarke error grid, Bland–Altman plot, mean absolute relative difference and glucose prediction error analysis were used to express point accuracy and the rate error grid to express trend accuracy. Reliability testing included aspects of the device and the external sensor, and the special central venous catheter (CVC) with a semipermeable membrane for use with this device.

**Results:**

We collected 594 paired values in 12 patients (65 [26–80; 8–97] (median [IQR; total range]) paired values per patient). Point accuracy: 93.6 % of paired values were in zone A of the Clarke error grid, 6.4 % were in zone B; bias was 4.1 mg/dL with an upper limit of agreement of 28.6 mg/dL and a lower level of agreement of −20.5 mg/dL in the Bland–Altman analysis; 93.6 % of the values ≥75 mg/dL were within 20 % of the reference values in the glucose prediction error analysis; the mean absolute relative difference was 7.5 %. Trend accuracy: 96.4 % of the paired values were in zone A, and 3.3 and 0.3 % were in zone B and zone C of the rate error grid. Reliability: out of 16 sensors, 4 had to be replaced prematurely; out of 12 CVCs, two malfunctioned (one after unintentional flushing by unsupervised nurses of the ports connected to the internal microdialysis chamber, causing rupture of the semipermeable membrane; one for an unknown reason). Device start-up time was 58 [56–67] min; availability of real-time data was 100 % of the connection time.

**Conclusions:**

In this study in critically ill patients who had no hypoglycemic episodes and a limited number of hyperglycemic excursions, point accuracy of the device was moderate to good. Trend accuracy was very good. The device had no downtimes, but 4 out of 16 external sensors and 2 out of 12 CVCs had practical problems.

**Electronic supplementary material:**

The online version of this article (doi:10.1186/s13613-016-0171-3) contains supplementary material, which is available to authorized users.

## Background

Most if not all critically ill patients receive intravenous infusion of insulin for blood glucose control at some point during stay in the intensive care unit (ICU) [1]. This strategy requires frequent blood glucose measurements for the guidance of insulin titrations, but this is both time- and blood-consuming [2]. Automation of blood sampling and glucose measurement through continuous glucose monitor (CGM) devices could reduce this burden and has the potential to improve overall blood glucose control [3, 4].

Microdialysis offers the opportunity to sample blood analytes with high accuracy but without the need for drawing blood samples. EIRUS^®^ (Maquet Critical Care AB, Solna, Sweden), a microdialysis-based device that can measure blood glucose and lactate levels, has been tested and validated previously in studies in surgical patients, where it has been found to be safe and accurate [4–7]. To date, its accuracy with regard to blood glucose monitoring, and reliability have not yet been tested extensively in ICU patients [4, 6].

We hypothesized the EIRUS^®^ system to be point and trend accurate and to be reliable in ICU patients. To test this hypothesis, we used this CGM device in a series of critically ill patients, to compare device readings with frequently measured arterial blood glucose values. Along the study, we determined reliability of the device and the special central venous catheter (CVC) with a semipermeable membrane designed for use with this device.

## Methods

### Study design and population

This investigator-initiated prospective study was conducted in the mixed medical-surgical ICU of the Academic Medical Center, Amsterdam, The Netherlands. The Institutional Review Board of the Academic Medical Center approved the study protocol, and informed consent was obtained from all patients or their legal representatives before start of the study. Maquet Critical Care AB provided the CGM device and its disposables free of charge. Maquet Critical Care AB had influence neither on the design of this study nor on reporting of the results. The study was registered at the Netherlands Trial Register (NTR4527).

Patients were eligible for participation if they were at least 18 years old, were expected to stay in the ICU for ≥48 h, had an arterial catheter in place and were in need of a (new) CVC. Patients were excluded if they participated in another investigational drug or device study or were known to be pregnant.

### Blood glucose control

ICU nurses followed a local guideline aiming at a blood glucose level between 90 and 144 mg/dL (5–8 mmol/L) as part of standard care. This guideline mandated nurses to measure blood glucose every 4 h, or more frequently when glucose levels were out of range or when rapid changes were expected. Infusion of insulin was started when glucose levels were over 144 mg/dL and stopped when glucose was lower than 61 mg/dL. Adjustments of insulin titration were based on sliding scales. More details can be found in Additional file 1. In addition, details on how nurses were trained can also be found in Additional file 1.

During the study, ICU nurses were not allowed to change insulin infusion rate based on the readings by the device. The ICU nurses, however, had access to device readings and additional arterial blood glucose measurements were allowed if the device suggested rapid changes in the glucose level or when there was a trend toward hypoglycemia. In addition, the ICU nurses could also adjust insulin infusion rates based on reference blood glucose values obtained during study observation periods (see below).

### The study device

For intravenous microdialysis-based glucose monitoring, a special CVC with a semipermeable membrane (Maquet Critical Care AB, Solna, Sweden) is needed. This CVC has five lumens, three ‘normal’ ports for intravenous administration of fluids or medication and two ‘special’ ports for transport of normal saline alongside the semipermeable membrane, which should not be flushed and cannot be used for intravenous administration of fluids or medication. The ‘afferent’ port is connected to a saline-filled syringe placed in the syringe pump of the device. The ‘efferent’ port is connected to the disposable sensor. Small metabolites such as glucose pass through the semipermeable membrane creating equilibrium between blood and the dialysate. The dialysate is pumped over the sensor in a continuous fashion, where the glucose oxidase method is used to measure the glucose level [4, 5]. The device can be used for a maximum of 96 h per sensor. Reference measurements are needed for calibration of the device, which is performed at start-up and every 8 h thereafter.

Of note, because the dialysate needs to be transported to the sensor outside the patient, where measurements are performed, there is a delay in time of 5 min between dialysate formation and the actual measurements.

### Study procedures

In three blocks of 8 h per day, and for a maximum of 3 days, every 15 min an arterial blood sample of 200 µL was drawn through an existing arterial line. Blood glucose levels were measured using a blood gas analyzer (RAPIDLab 1265, Siemens Healthcare Diagnostics, The Hague, The Netherlands).

Definitions of the metrics used to assess device reliability, including those suggested by recent consensus recommendations [8], are described in detail in Additional file 1 and included the percentage of real-time data, skips in data acquisition, failures to calibrate, sensor failures and CVC failures.

### Power calculation

Based on previous studies [9, 10], we chose to collect approximately 1000 paired measurements or to connect the device to a minimum number of 11 patients. Inclusion of patients was restricted by the time the device was available for this study and the number of disposable CVCs and sensors provided by the manufacturer.

### Analysis plan

Patient characteristics were reported as means, medians or percentages, where appropriate. Because of the delay between dialysate formation and the actual measurements of the blood glucose level, we subtracted 5 min from the time stamp of the values of the CGM device; as such reference blood glucose values matched with the moment dialysate was formed. Subsequently, device and reference measurements were merged. Paired measurements were used for determining point and trend accuracy of the device. To be considered for the statistical analysis, each patient needed to have at least multiple samples with at most 30 min in between. However, patients excluded for statistical analysis remained included in the reliability analysis. While each paired sample was included in the point accuracy analysis, only the samples with a gap of at most 30 min to the next sample were included in the trend accuracy analysis. In addition, when the device was calibrated within the daily 8-h block of intense sampling, the calibration sample and the subsequent sample were not considered for trend accuracy analysis. This way, large changes in trend due to the calibration were excluded from the analysis.

Point accuracy was expressed using a Clarke error grid, a Bland–Altman plot, the glucose prediction error analysis and the mean absolute relative difference (MARD). To be considered point accurate, at least 95 % of values must be in zone A, a maximum of 5 % can be in zone B, and no values are allowed in zones C to E of the Clarke error grid [11]. Also, the MARD should be below 14 %; a value above 18 % represent poor accuracy [3].

Trend accuracy was expressed using rate error grid analysis (R-EGA) [12]. Values outside zones A and B of the R-EGA corresponding to values in zones A and B of the Clarke error grid were considered benign errors. On the other hand, values outside zones A and B of the R-EGA corresponding to values outside zones A and B of the Clarke error grid were considered erroneous readings [12].

### Post hoc analysis

Point accuracy was also expressed using the recently published surveillance error grid [13].

Two of the CVCs were malfunctioning. In one case, it was immediately clear that the CVC was defect, and no additional measurements were performed. In another case, this was not immediately clear, and only after reviewing the readings it became clear that the CVC started to malfunction from a certain time point. We chose to perform a post hoc analysis excluding the data from that patient.

## Results

**Fig. 1:**
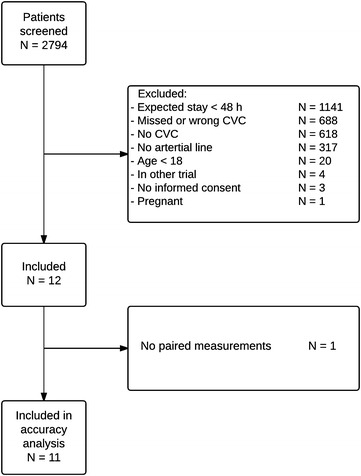
Consolidated standards of reporting trials (CONSORT) diagram

A total of 12 patients were included in whom 598 paired measurements were available. Figure 1 shows the CONSORT diagram. One patient was excluded from the point and trend accuracy analyses because no comparative samples could be obtained while the device was connected due to calibration problems. In one patient, four arterial blood samples had to be discarded as they were diluted during sampling. Thus, we had 594 samples (65 [26–80; 8–97] (median [IQR; total range]) paired values per patient) for determining point accuracy of the CGM device. For trend accuracy analysis, 482 samples were used. Patient characteristics are shown in Table 1. Metrics of glucose control are shown in Table 2.

**Table 1:** Patient characteristics

Age in years, median [IQR])	65 [60–79]
Male gender, number (%)	6/50 %
Race, number (%)	
Caucasian	10 (83.3 %)
Black	1 (8.3 %)
Asian	1 (8.3 %)
BMI in kg/m^2^, median [IQR]	23 [20–27]
Admission type, number (%)	
Medical	6 (50 %)
Emergency surgery	3 (25 %)
Planned surgery	3 (25 %)
History of diabetes, number (%)	
No diabetes	11 (92 %)
Diabetes, unknown treatment	1 (8 %)
Diabetes treated with insulin	0 (0 %)
Diabetes treated with oral agents	0 (0 %)
APACHE II, median [IQR]	21 [18–26]
SAPS II, median [IQR]	44 [37–53]
ICU LOS, days, median [IQR]	15 [7–17]
Hospital LOS, days, median [IQR]	20 [18–35]
ICU mortality, number (%)	7 (58 %)
Hospital mortality, number (%)	8 (67 %)

*IQR* interquartile range, *BMI* body mass index, *APACHE II* acute physiology and chronic health evaluation II, *SAPS II* sepsis-related organ failure assessment score II, *ICU* intensive care unit, *LOS* length of stay

**Table 2:** Metrics of glucose control

Total number of measurements	594
Mean blood glucose level per patient, mg/dL, median [IQR; total range]	133 [118–140; 112–162]
Standard deviation of blood glucose level per patient, mg/dL, median [IQR; total range]	15 [11–18; 1–49]
Number of measurements per patient, median, [IQR; total range]	65 [26–80; 8–97]
Mild hyperglycemia 150–179 mg/dL in measurements, number (%)	62 (10)
Mild hyperglycemia 150–179 mg/dL in patients, number (%)	10 (91)
Severe hyperglycemia >180 mg/dL in measurements, number (%)	29 (5)
Severe hyperglycemia >180 mg/dL in patients, number (%)	3 (27)
Severe hypoglycemia ≤40 mg/dL in measurements, number (%)	0
Severe hypoglycemia ≤40 mg/dL in patients, number (%)	0
Mild hypoglycemia 41–70 mg/dL in measurements, number (%)	0
Mild hypoglycemia 41–70 mg/dL in patients, number (%)	0

*IQR* interquartile range

### Point and trend accuracy

**Fig. 2:**
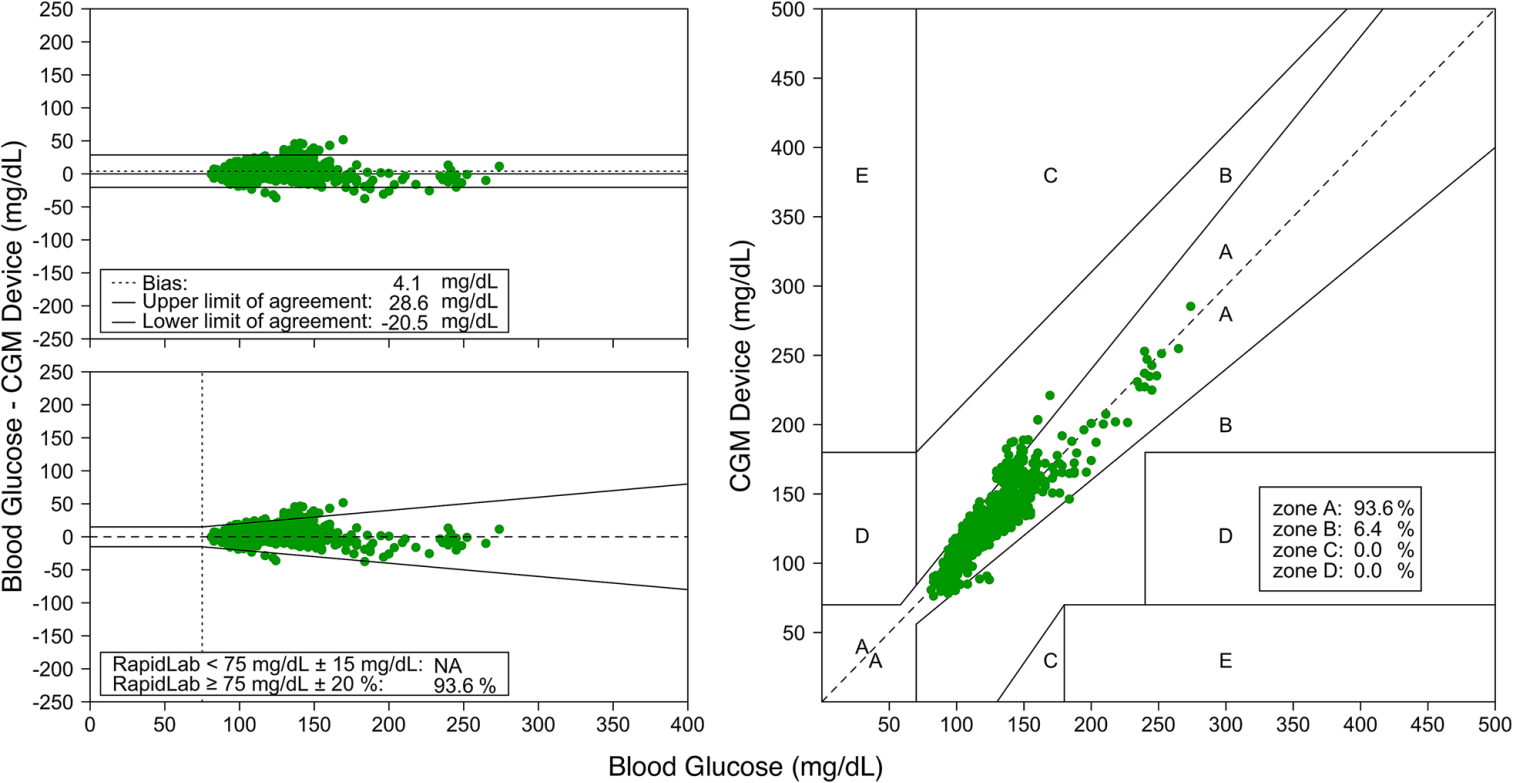
Measures of point accuracy. Bland–Altman plot (*upper left panel*), glucose prediction error grid (*lower left panel*) and Clarke error grid (*right panel*)

**Fig. 3:**
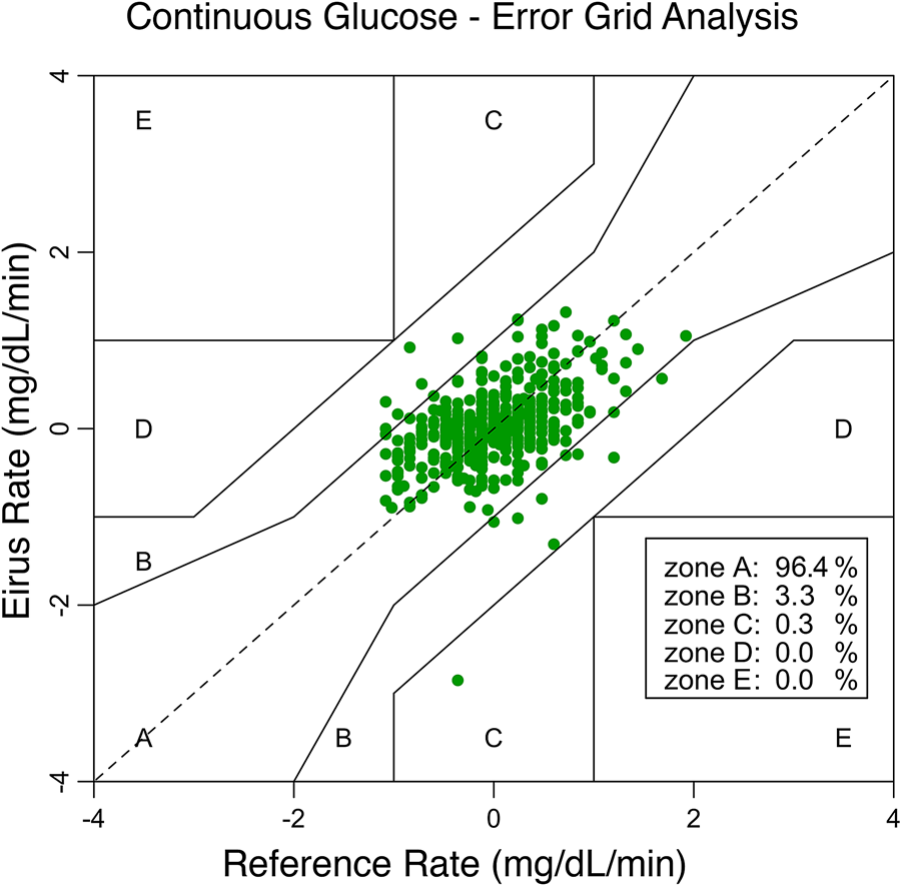
Rate error grid of the continuous glucose error grid analysis. This grid is divided into similar zones as the Clarke error grid. Perfectly trend accurate values are the *dashed line* in the middle

The Clarke error grid, Bland–Altman plot and glucose prediction error grid are presented in Fig. 2. Bias in the Bland–Altman plot was 4.1 mg/dL with an upper limit of agreement of 28.6 mg/dL and a lower limit of agreement of −20.5 mg/dL. Glucose prediction error analysis showed that 93.6 % of the values ≥75 mg/dL within twenty percent of the values measured by the blood gas analyzer were within range. The MARD was 7.5 %. The rate error grid is presented in Fig. 3, consisting of 99.7 % accurate readings and 0.3 % benign errors.

### Reliability

Table 3 shows reliability results. Start-up time was 58 [56–67; 48–112.8] (median [IQR; total range]) min. In three patients, the initial sensor could not be calibrated at start-up, and a second sensor was needed. In two patients, the CVC malfunctioned after some hours. In one patient, this was due to improper handling by one of the trained ICU nurses. This and other details on reliability are discussed in more detail in Additional file 1.

**Table 3:** Reliability and safety of the CGM device

	In total
Total number of sensors used	16

	Per patient
Number of sensors used, median [IQR; total range]	1.0 [1.0–2.0; 1–2]
Total connection time, median [IQR; total range]	50.8 [13.5–54.7; 2.5–55.8] h
Real-time data, median [IQR; total range] (%)	50.8 [13.5–54.7; 2.5–55.8] h (100 %)
Time of skips in data acquisition	0.0 h
Percentage of time skips in data acquisition	0.0 h
Initialization time, median [IQR; total range]	42 [42–43; 40.8–62.4] min
Total start-up time, median [IQR; total range]	58 [56–67; 48–112.8] min
Number of calibrations needed before start, median [IQR; total range]	1.0 [1.0–1.3; 1–3]
Number of calibrations during duration of measurement, median [IQR; total range]	6.5 [2.0–7.0; 1–8]
Number of failed calibrations during duration of measurement, median [IQR; total range]	0.5 [0.0–2.0; 0–3]

IQR, interquartile range; initialization time, total time from connecting the device, to being ready for calibration; total start-up time, total time from connecting the device, to displaying the first glucose value

### Post hoc analysis

The surveillance error grid is presented in Additional file 1: Figure S1.

Results of the post hoc analysis excluding the data from the patient mentioned above in whom the CVC was malfunctioning for unknown reasons is presented in Additional file 1: Figure S2, Figure S3, Figure S4 and Figure S5.

## Discussion

In this study in a cohort of critically ill patients, point accuracy of a microdialysis-based CGM device developed was moderate to good. Trend accuracy was very good. Reliability was moderate, seen as 4 out of 16 external sensors could not be used and 2 out of 12 CVCs had practical problems.

Point accuracy in the present study was less than the point accuracy reported from two previous studies in patients after cardiac surgery [4, 6]. In these studies, all paired values were in zones A and B, with 97 and 99 % of values in zone A of the Clark error grid, and the MARD was only 5.6 and 5 %, respectively. Both those studies and the present study used arterial blood gas analyzers as a reference standard. The present study, however, was conducted in patients that were more severely ill than cardiac surgery patients, reflected by a longer length of stay in the ICU stay (15 vs. 3 days) and hospital (20 vs. 8 days). Thus, these two studies included completely different patients, which could at least in part explain the differences. The results of the present study, however, are very similar to a pilot study in abdominal surgery patients [5], in which all paired values were in zones A and B, with 94 % of values in zone A of the Clark error grid.

According to a recent consensus on blood glucose monitoring, 95 % of paired values need to be in zone A of the Clarke error grid to qualify a device as point accurate [11]. In contrast, a more recent consensus among a panel of ICU experts, the MARD should be <14 % [3]. While the studied device did not meet the first criteria, it did meet the last. There are no generally accepted criteria for trend accuracy of CGM devices in the ICU setting [3]. Nonetheless, we believe EIRUS to be very accurate, as only one value was in the benign error range [12]. In addition, it should be noted that the paired measurement in zone C mentioned above came from the patient in whom the special CVC was malfunctioning. Since both glucose and lactate measured by the device decreased rapidly and non-physiologically, we suspect that the semipermeable membrane of that CVC broke.

Both the afferent and efferent ports of the CVC, connected to the dialysate chamber, were labeled with tags mentioning not to flush these ports. Unfortunately, unsupervised nurses thought there was backflow of blood in the afferent port because of the deep-red/purple color and flushed it with normal saline immediately. This resulted in a rupture of the delicate semipermeable membrane and thus malfunctioning of the CVC: Saline pumped into the chamber disappeared into the circulation, and the efferent port stopped producing dialysate. After this we continued using the special CVC as a normal CVC, with two stops at the extra ports. The manufacturer changed the color of the lumen and its connector to prevent this incident after this study. These problems, however, did raise some concerns. However, we do not believe that these problems were caused by an insufficient introduction of the study device in the unit since we organized multiple training sessions for nurses and instructed nurses individually when a patient was included in the study and monitored by the device.

Our study has several limitations. First and most importantly, no hypoglycemic periods were captured during the study, and the number of hyperglycemic events was small. While the device proved point accurate in the hypoglycemic range in one study in animals [14], we remain uncertain on hypoglycemic performance in ICU patients. The absence of hypoglycemia might be explained by the fact that reference measurements were performed very frequently and because nurses had access to the device readings. Nurses were allowed to use the reference measurements and thus could improve blood glucose control (i.e., prevent hypoglycemic events). Even the device readings could have helped nurses to prevent dangerous excursions of the blood glucose level, even though they knew that this was an investigational device. The local Institutional Review Board did not accept blinding the nurses for the reference measurements and the device readings. In addition, the fact that we only actively collected paired measurements during daytime hours means that we might have missed possible interesting data overnight. More paired samples, also outside working hours, could have yielded more hypoglycemic events. To make a more conclusive statement on device accuracy in the hypoglycemic range, other methods for capturing hypoglycemic and hyperglycemic events have to be further explored. One recently suggested way to improve the execution of accuracy testing of investigational devices in the clinical setting includes data mining of electronic medical records [15, 16]. Data mining is a technique that uses large quantities of data in search for certain events, in this case hypoglycemia and hyperglycemia. Comparison between consecutive measurements of the blood glucose level by means of a CGM device and comparative measurements in a central laboratory then could be used to determine the accuracy in these extreme situations. This approach certainly increases the number of hypoglycemic and hyperglycemic events that can be used for accuracy testing, but of course requires extensive use in one of more intensive care units. Finally, as of September of 2015, shortly after analyzing the data before reaching our goal of 1000 paired samples, we had to stop the study prematurely. The data allowed for a sufficiently narrow interval of confidence on the point and trend accuracy of the machine and therefore we did not consider it ethically justified to include more patients, seen the potential burden and risks of obtaining blood samples every 15 min.

There were also several strengths to this study. This is the first study to date to investigate trend accuracy of a CGM device in critically ill patients. In addition, the investigated microdialysis CGM device had not been tested in a mixed ICU before. This makes the results of this study more clinically applicable as this is indeed the patient population in which glucose monitoring is most relevant. Finally, we used precise blood gas analyzers for reference measurements, and we corrected for the 5-min delay between formation of the dialysate and the measurement at the sensor side.

## Conclusion

The point and trend accuracy of the tested microdialysis-based CGM device was moderate to good in patients who were stable with regard to their blood glucose levels. Trend accuracy was very good. The device had no downtimes, but 4 out of 16 external sensors and 2 out of 12 CVCs had practical problems.

## Additional file

10.1186/s13613-016-0171-x Surveillance error grid with risk scores. **Figure S2.** Blood glucose, CGM and lactate values of patient with failing CVC. **Figure S3.** Measures of point accuracy. Bland-Altman plot (upper-left panel), glucose prediction error grid (lower-left panel) and clarke error grid (right panel). **Figure S4.** Rate Error–Grid of the Continuous glucose–error grid analysis. This grid is divided into similar zones as the Clarke error grid. Perfectly trend accurate values are the dashed line in the middle. **Figure S5.** Post-hoc Surveillance error grid with risk scores.
