# Supplementary material for: Point and trend accuracy of a continuous intravenous microdialysis-based glucose-monitoring device in critically ill patients: a prospective study
Source: Ann Intensive Care. 2016 Jul 19;6:68. doi: 10.1186/s13613-016-0171-3 (PMC4951389; doi:10.1186/s13613-016-0171-3)
Supplement: Supplementary file 1 — 10.1186/s13613-016-0171-x Surveillance error grid with risk scores. Figure S2. Blood glucose, CGM and lactate values of patient with failing CVC. Figure S3. Measures of point accuracy. Bland-Altman plot (upper-left panel), glucose prediction error grid (lower-left panel) and clarke error grid (right panel). Figure S4. Rate Error–Grid of the Continuous glucose–error grid analysis. This grid is divided into similar zones as the Clarke error grid. Perfectly trend accurate values are the dashed line in the middle. Figure S5. Post-hoc Surveillance error grid with risk scores. [file 13613_2016_171_MOESM1_ESM.docx]

Additional file 1 to:

**Point and Trend Accuracy of a Continuous Intravenous Microdialysis–based Glucose–monitoring Device in Critically Ill Patients – a Prospective Study**

J.H. Leopold^1,2^, R.T.M. van Hooijdonk^1^, M. Boshuizen^1^, T. Winters^1^,

L.D. Bos^1^, A. Abu–Hanna^2^, A.M.T. Hoek^3^, J.C. Fischer^3^, E.C van Dongen-Lases^3^

M.J. Schultz^1,4^

**Academic Medical Center, Amsterdam, The Netherlands:**

^1^Department of Intensive Care

^2^Department of Medical Informatics

^3^ Department of Clinical Chemistry

^4^Laboratory of Experimental Intensive care and Anesthesiology (L.E.I.C.A)

**Correspondence**

Jan Hendrik Leopold

Academic Medical Center

Room C3–311

Meibergdreef 9

1105 AZ Amsterdam

The Netherlands

E–mail: [j.h.leopold@amc.uva.nl](mailto:j.h.leopold@amc.uva.nl)

**Email addresses other authors:**

Roosmarijn T.M. van Hooijdonk [r.t.vanhooijdonk@amc.uva.nl](mailto:r.t.vanhooijdonk@amc.uva.nl)

Margit Boshuizen [m.boshuizen@amc.uva.nl](mailto:m.boshuizen@amc.uva.nl)

Tineke Winters [t.winters@amc.uva.nl](mailto:t.winters@amc.uva.nl)

Lieuwe D.J. Bos: [l.d.bos@amc.uva.nl](mailto:l.d.bos@amc.uva.nl)

Ameen Abu-Hanna: [a.abu-hanna@amc.uva.nl](mailto:a.abu-hanna@amc.uva.nl)

Arthur M.T. Hoek [a.m.hoek@amc.uva.nl](mailto:a.m.hoek@amc.uva.nl)

Johan C. Fischer: [j.c.fischer@amc.uva.nl](mailto:j.c.fischer@amc.uva.nl)

Edmée C. van Dongen-Lases:  [e.c.vandongen-lases@amc.uva.nl](mailto:a.abu-hanna@amc.uva.nl)

Marcus J. Schultz: [marcus.j.schultz@gmail.com](mailto:marcus.j.schultz@gmail.com)

# Supplemental information

## Guideline for glucose control

A local guideline aiming at a blood glucose level between 90–144 mg/dL (5–8 mmol/L) was followed as part of standard care with insulin infusion being initiated when glucose levels rose over 144 mg/dL. When glucose levels were below 61 mg/dL insulin infusion was stopped and boluses of dextrose were given. Sliding scales were used for the adjustment of insulin titration. Insulin was only infused intravenously and in a continuous manner and boluses were exclusively administered when blood glucose levels rose over 360 mg/dL.

The guideline mandated nurses to measure blood glucose at least every four hours, or more frequently when glucose levels were out of range or when rapid changes were expected. Blood gas analyzers (RAPIDLab 1265, Siemens Healthcare Diagnostics, The Hague, The Netherlands) were used to analyze the samples. Data was stored in the patient data management system.

*Training of the nurses and the use of the device*

Before the study device was introduced into the unit, nurses were trained on how to use and calibrate the device. When a patient was eligible for inclusion in the study and after placement of the special CVC was placed the device was connected by the researchers who performed the first calibrations. Nurses were instructed on how to use the CVC, in particular not to flush the two special ports used by the CGM device. In addition, both ports were labeled with adhesive tags clearly showing the following text: ‘DO NOT FLUSH’ (in Dutch: ‘NIET FLUSHEN’).

## Metrics for device reliability

- Total number of sensors used – Total number of sensors used between all patients.
- Number of sensors used – Number of sensors used per patient.
- Total connection time – Total connection time the system was connected to a patient.
- Real–time data – Total time the data was displayed in real-time.
- Time of skips in data acquisition – Time the data was not displayed.
- Percentage of time skips in data acquisition – Percentage of the time the data was not displayed.
- Initialization time – Total time from connecting the device, to being ready for calibration.
- Total start-up time - Total time from connecting the device, to displaying the first glucose value.
- Number of calibrations needed before start – Number of calibrations that were necessary before first glucose value was displayed.
- Number of calibrations during duration of measurement – Total number of calibrations that were performed during the time the device was connected to the patient.
- Number of failed calibrations during duration of measurement – Number of calibrations that failed during the time the device was connected to the patient.

**Reliability and practical problems**

In three patients, the initial sensor could not be calibrated at start–up, and a second sensor was needed. In one patient, the device gave repeated calibration problems, also after replacing the sensor. No measurements could be taken in this patient. This is the aforementioned patient that was excluded from the point and trend accuracy analysis. Calibrated sensors had no down–times and displayed values for 100% of the connection time. In one patient, a non–supervised but trained ICU nurse unintentionally flushed the afferent port of the CVC, causing an abrupt rupture of the semipermeable membrane, and consequently this CVC could no longer be used for continuous blood glucose monitoring (data from this patient were excluded from the point and trend accuracy analysis). In another patient the CVC malfunctioned for an unknown reason. While device data showed a stop in flow, possibly caused by membrane rupture, the nurse denied flushing the special ports of the CVC. Figure S2 shows that the trend in blood glucose levels was comparable to that of blood lactate levels. The non–physiologic drop in lactate allowed us to identify malfunctioning of the CVC, for yet unknown reasons.

# Post-hoc analysis excluding data from one patient with a malfunctioning CVC

## Methods

To investigate if the special CVC was malfunctioning in this patient, we analyzed both continuous glucose and lactate data as measured by EIRUS^®^ system and plotted the values against reference values from our blood gas analyzer.

To investigate the point and trend accuracy of EIRUS^®^ system without the patient in whom the special CVC was malfunctioning, data from the aforementioned patient was excluded. Thereafter, the same instruments were used to analyze point – and trend accuracy. Point accuracy was expressed using a Clarke error grid, a Bland–Altman plot, the glucose prediction error analysis, the mean absolute relative difference (MARD) and the Surveillance error grid. Trend accuracy was expressed using rate error grid Analysis (R–EGA)

## Results

As can been seen in figure S2, both glucose and lactate values as measured by the EIRUS^TM^ drop significantly within 15 minutes (133 mg/dL to 90 mg/dL and 23 mg/dL to 17 mg/dL, respectively). We consider this fast change to be implausible. In addition, the monitor intermittently displayed the message that it was flushing the line. Therefore, we suspect that the CVC was not performing correctly, possibly after being flushed.

A total of 582 paired measurements in 10 patients were analyzed. The Clarke error grid, Bland–Altman plot, and glucose prediction error grid of the post-hoc analysis are presented in Figure S3. Bias in the Bland–Altman plot was 4.0 mg/dL with an upper limit of agreement of 28.0 mg/dL and a lower limit of agreement of -19.9 mg/dL. Glucose prediction error analysis showed that 94.3% of the values ≥ 75 mg/dL within twenty percent of the values measured by the blood gas analyzer were within range. The MARD was 7.3%. The rate error grid is presented in Figure S4. The Surveillance error grid is presented in figure S5.

**Figures**

Figure S1. Surveillance error grid with risk scores.


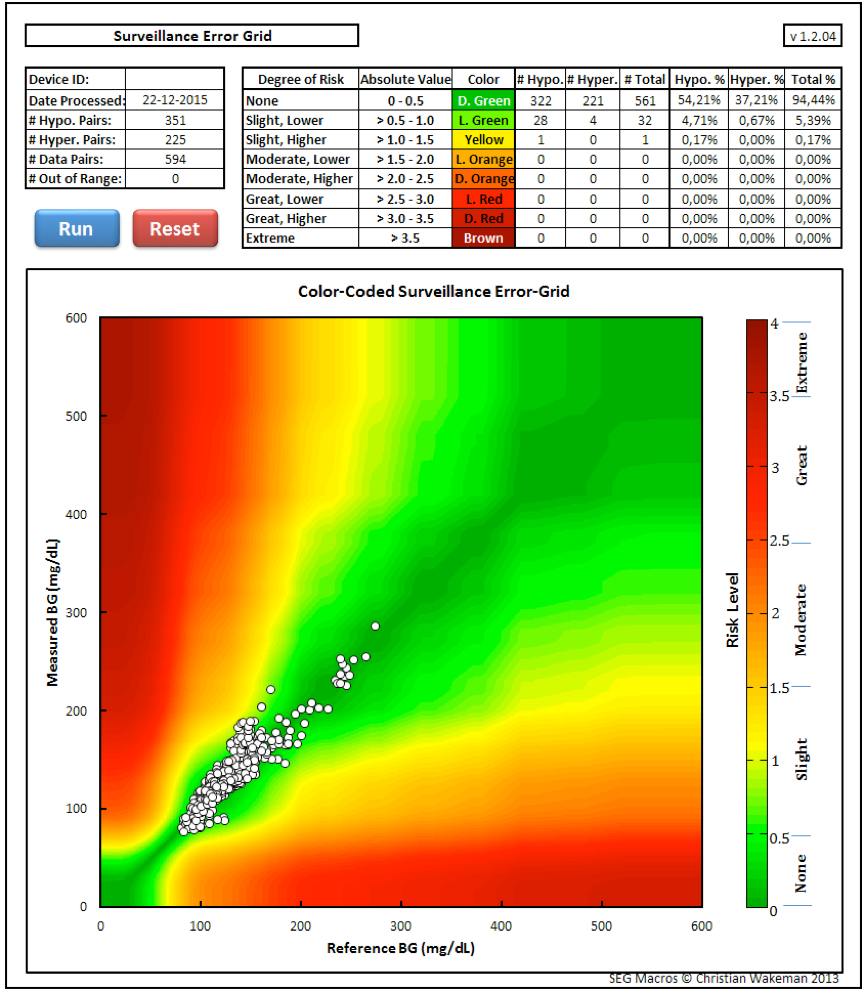


Figure S2: Blood glucose, CGM and lactate values of patient with failing CVC


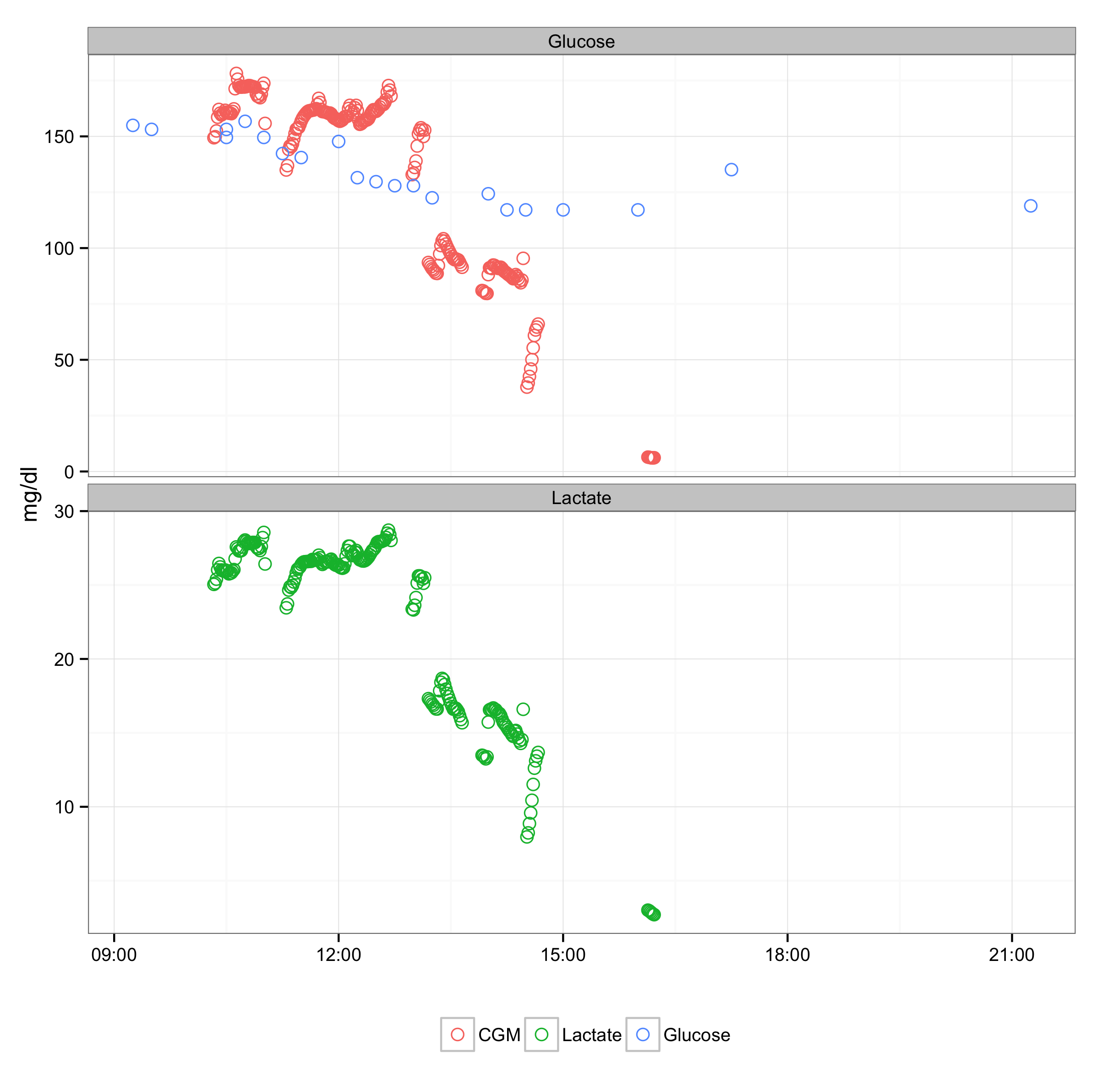


Figure S3. Measures of point accuracy. Bland-Altman plot (upper-left panel), glucose prediction error grid (lower-left panel) and clarke error grid (right panel).


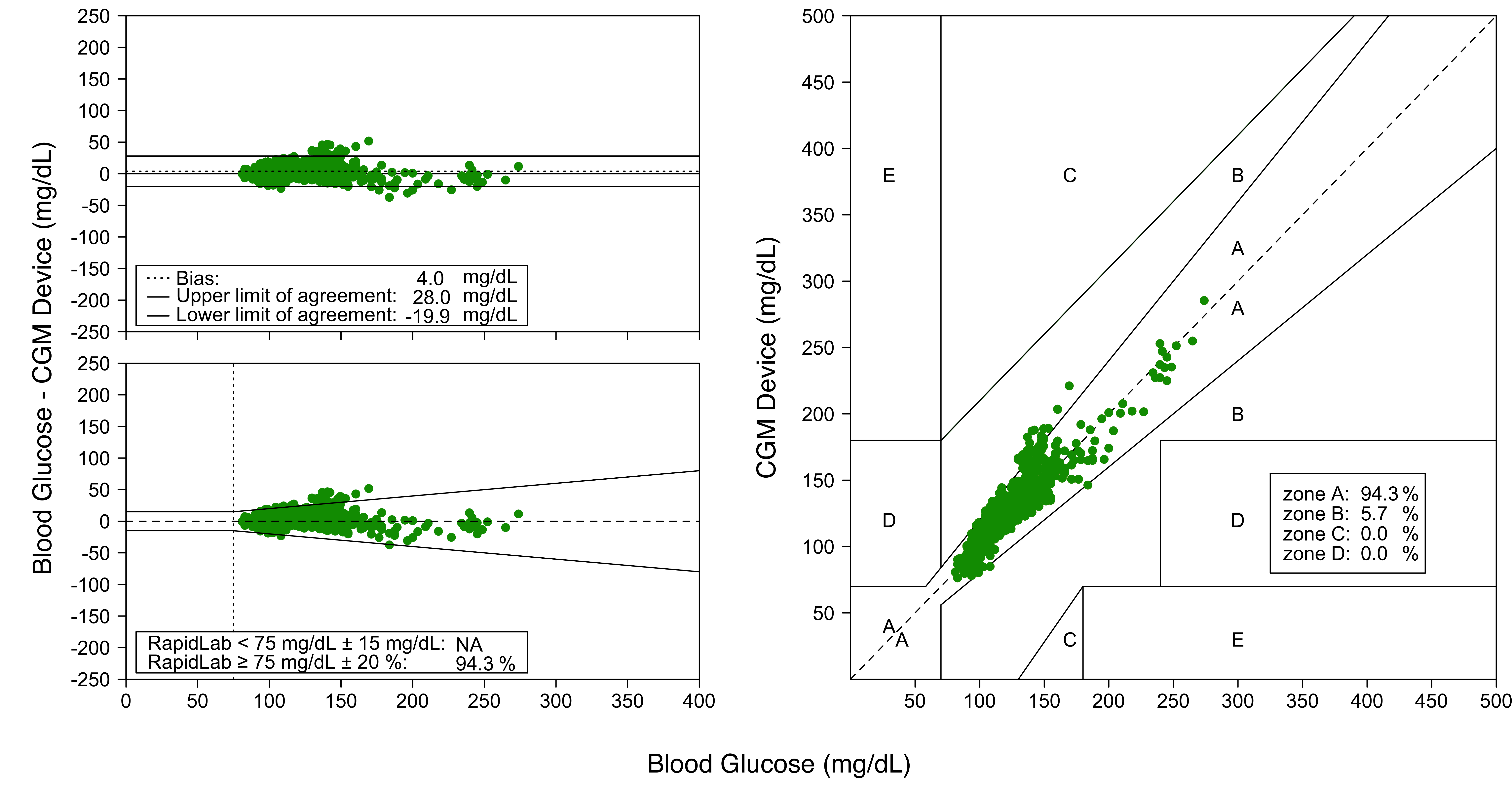


Figure S4. Rate Error–Grid of the Continuous glucose–error grid analysis This grid is divided into similar zones as the Clarke error grid. Perfectly trend accurate values are the dashed line in the middle.


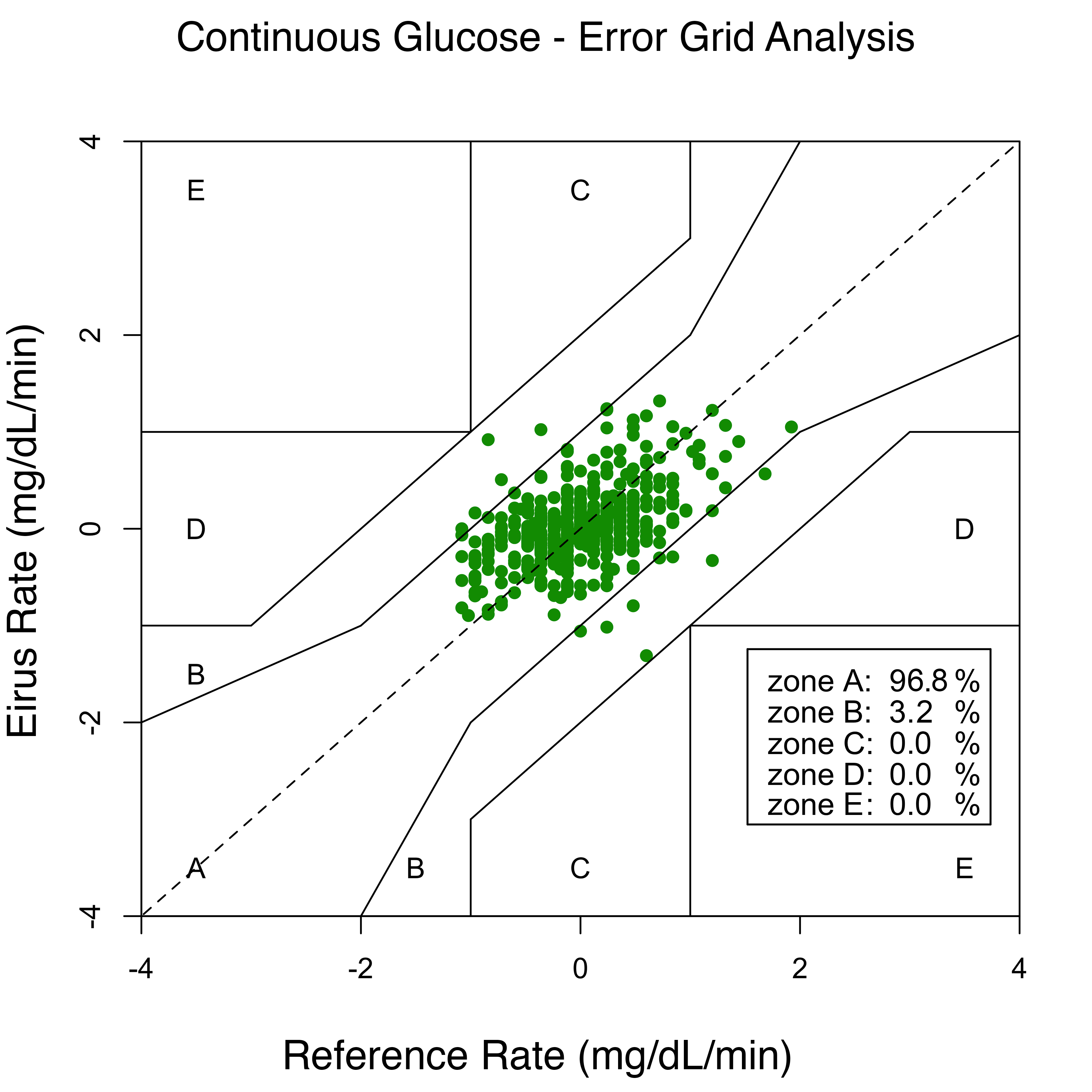


Figure S5. Post-hoc Surveillance error grid with risk scores.

**
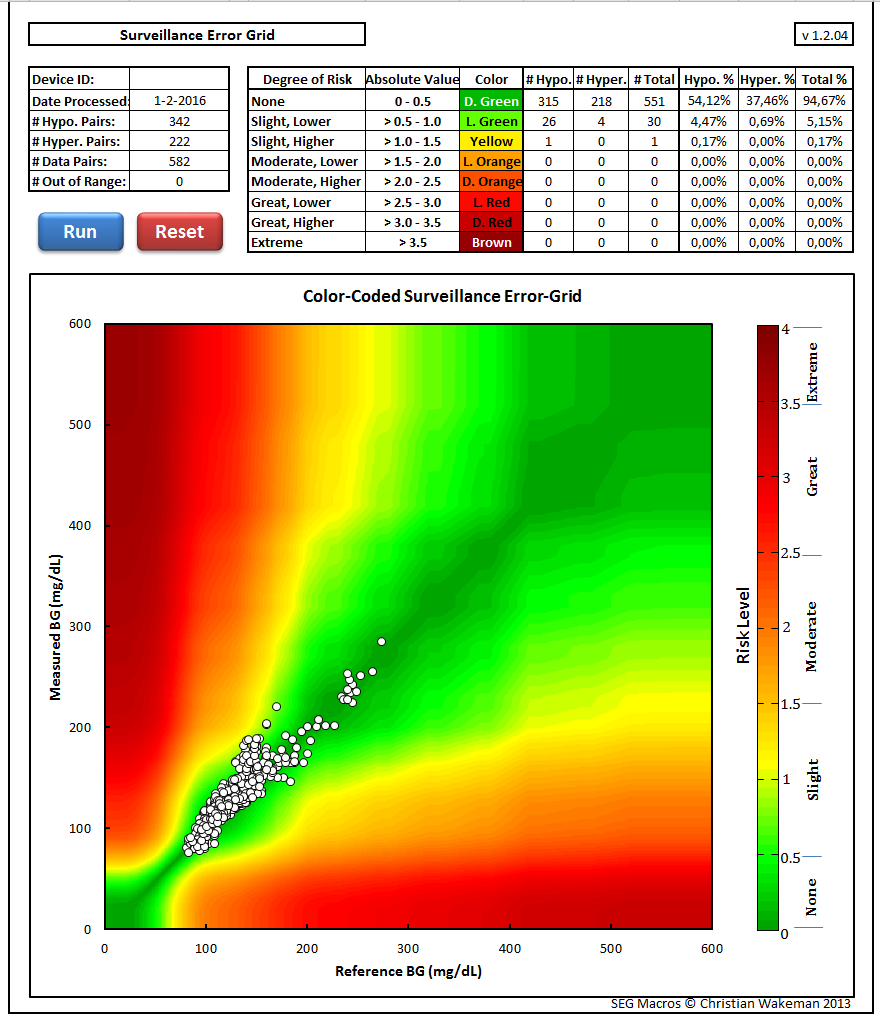
**
